# Single-Nucleotide Polymorphisms of BRCA1 and BRCA2 and Risk of Papillary Thyroid Carcinoma

**DOI:** 10.3390/cancers17091456

**Published:** 2025-04-26

**Authors:** Chang Myeon Song, Yun Jin Kim, Hyun Sub Cheong, Yong Bae Ji, Kyung Tae

**Affiliations:** 1 Department of Otolaryngology-Head and Neck Surgery, College of Medicine, Hanyang University, Seoul 04763, Republic of Korea; jyb20000@hanmail.net; 2 Department of Pre-Medicine, College of Medicine, Hanyang University, Seoul 04763, Republic of Korea; yeun0148@hanyang.ac.kr; 3 Biostatistics Lab, Medical Research Collaborating Center, Hanyang University, Seoul 04763, Republic of Korea; 4 Agro SP Inc., 244 Beotkkot-ro, Geumcheon-gu, Seoul 08513, Republic of Korea; chhs@agrosp.co.kr

**Keywords:** breast cancer gene, BRCA1, BRCA2, single-nucleotide polymorphism, papillary thyroid carcinoma, genetic polymorphism

## Abstract

**Background/Objectives**: We sought to evaluate the association between the risk of papillary thyroid carcinoma (PTC) and single-nucleotide polymorphisms (SNPs) of breast cancer genes 1 (*BRCA1*) and 2 (*BRCA2*). **Methods**: We prospectively recruited 515 cases with PTC and 296 controls without cancer. The genotypes of five *BRCA1* SNPs (rs8176318, rs1799966, rs799917, rs16940, rs1799949) and three *BRCA2* SNPs (rs15869, rs1799943, rs1799955) were determined using the TaqMan assay. We evaluated the association of haplotypes with the risk of PTC due to linkage disequilibrium (LD). **Results**: The five *BRCA1* SNPs were significantly associated with the risk of PTC. The AC genotype of rs8176318 (OR = 0.69, *p* = 0.02) and the CT and CC genotypes of rs1799966 (OR = 0.70, *p* = 0.02 and OR = 0.67, *p* = 0.01, respectively) were associated with a decreased risk of PTC. The AG genotype of rs16940 (OR = 0.67, *p* = 0.01) and the AG genotypes of rs799917 and rs1799949 (both OR = 0.70, *p* = 0.02) decreased the risk of PTC. Haplotype 1 [rs8176318(C)-rs1799966(T)-rs799917(G)-rs16940(A)-rs1799949(G)] ± (OR = 0.69, *p* = 0.02) and haplotype 2 [rs8176318(A)-rs1799966(C)-rs799917(A)-rs16940(G)-rs1799949(A)] ± (OR = 0.71, *p* = 0.03) of *BRCA1* reduced the risk of PTC. **Conclusions**: Our findings suggest that the polymorphisms of *BRCA1* may contribute to the susceptibility to PTC in the Korean population.

## Simple Summary

This study was conducted to analyze the association between genetic polymorphisms of breast cancer genes 1 (*BRCA1*) and 2 (*BRCA2*), which are well known for their association with breast cancer and the development of thyroid cancer. We analyzed the relationship between single-nucleotide polymorphisms (SNPs) in *BRCA1* and *BRCA2* and thyroid cancer and conducted a haplotype analysis of these polymorphisms to study the interactions between genetic polymorphisms. The results of this study show that polymorphisms of BRCA1 were significantly related to the susceptibility to papillary thyroid carcinoma in the Korean population.

## 1. Introduction

Thyroid cancer includes differentiated carcinoma, such as papillary and follicular carcinoma, as well as non-differentiated cancers, including medullary, anaplastic carcinoma, and lymphoma. Thyroid cancer accounts for approximately 2% of all types of cancer diagnosed worldwide, and 3.5–4% of those are diagnosed in the United States [1,2]. Its incidence rates are four times higher among females than males [3]. However, the cause of this gender difference is not well known.

Ionizing radiation is an established risk factor for differentiated thyroid cancer, and its association with genetic changes is well known [4]. The incidence of thyroid cancer increases with exposure to environmental radiation, including that caused by the Chernobyl nuclear power plant accident and the explosion of atomic bombs [5,6,7]. The latency period is at least 5 to 10 years, and molecular alterations, including gene rearrangements, are frequently implicated [8]. Experimental radiation exposure results in genetic changes and tumorigenicity in thyroid epithelial cell lines, supporting the evidence for the effect of radiation on genetic changes related to thyroid cancer [9]. Furthermore, the risk of thyroid cancer is elevated 8-fold in first-degree relatives of thyroid cancer patients [10]. Therefore, an investigation of the basis of genetic predisposition of thyroid cancer is needed to fully understand the pathogenesis of differentiated thyroid carcinoma. However, familial inheritance can be related to both genetic predisposition and a shared common environment. A nationwide cohort study of Asian patients with non-medullary thyroid cancer reported that genetic and common environmental contributions to first-degree relative thyroid cancer were 28% and 14%, respectively [11]. Relative risks for non-medullary thyroid cancer for siblings, offspring, and parents were 6.4, 5.2, and 5.1, respectively.

Studies of the association of genetic variation and thyroid cancer have included several genome-wide association studies [11,12,13,14,15]. Various single-nucleotide polymorphisms (SNPs) have been shown to be associated with the risk of non-syndromic differentiated thyroid carcinoma [16,17,18,19]. Genes related to thyroid cancer development are involved in DNA repair, ATM (ataxia–telangiectasia mutated) signaling, RET (receptor tyrosine kinase) signaling, and the regulation of thyroid activity (*FOXE1*, *NKX2-1*, *TSHR*) [7]. The DNA repair systems associated with thyroid cancer development are the direct repair, excision repair, recombinant repair, and mismatch repair pathways [20]. SNPs that are associated with radiation-related thyroid cancer and ionizing radiation exposure in thyroid cancer patients include *FOXE1*, *MGMT*, *ALKBH3*, *LIG1*, *XRCC2*, *NKX2-1*, *ATM*, and *TP53* [21,22,23,24,25].

*BRCA1* and *BRCA2* are tumor suppressor genes involved in DNA damage repair before cell division is initiated [26]. The *BRCA1* DNA repair-associated protein (BRCA1) is implicated in DNA damage repair and transcription repression and acts as a cell cycle checkpoint [27]. It also interacts with tumor suppressors, DNA damage repair proteins, oncogenes, transcriptional activators/repressors, and cell cycle regulators [27]. The BRCA1- and BRCA1-interacting protein C-terminal helicase 1 (BRIP1) complex plays an important role in double-strand break repair. Mutation of *BRCA1* results in abnormal apoptosis, cell cycle checkpoint defects, chromosome instability, aneuploidy, and impaired DNA damage repair [28]. In contrast, the *BRCA2* DNA repair-associated protein (BRCA2) regulates the intracellular shuttling and activity of RAD51, a recombinase enzyme active in DNA repair via homologous recombination [29].

Mutations in *BRCA1* and *BRCA2* are associated with a risk of breast, ovary, prostate, bladder, and pancreatic cancer, as well as melanoma and other skin cancers [30]. *BRCA1* mutations have also been identified in patients with double primary cancers of the breast and thyroid [31,32]. However, the association of *BRCA1*/*BRCA2* genetic polymorphism with the risk of papillary thyroid carcinoma (PTC) has not been thoroughly investigated. We aimed to prioritize the investigation of SNPs to facilitate the subsequent selection of additional genetic targets. In this study, we analyzed the single-nucleotide polymorphisms of *BRCA1/BRCA2* in patients with PTC and controls to assess the association of *BRCA1/BRCA2* polymorphism and the risk of PTC. To the best of our knowledge, this study is the first to examine the association between *BRCA1/BRCA2* polymorphism and the risk of PTC in the Korean population.

## 2. Materials and Methods

### 2.1. Subjects

We prospectively enrolled patients with pathologically confirmed PTC, who had undergone surgery at a tertiary hospital. Although both PTC and follicular carcinoma are classified as differentiated carcinomas, we limited our study to PTC in order to maintain homogeneity. The controls consisted of healthy volunteers who visited the hospital for health check-ups or patients who were admitted to the hospital for treatment of upper respiratory infections or for operations on benign lesions of the ear, nose, and throat. We included subjects over 18 years of age of Korean nationality who resided in South Korea. We excluded subjects with previous or simultaneous histories of malignancy other than thyroid cancer or histories of genetic disease. Patients who received radiation therapy were excluded. Finally, 811 subjects (515 patients with PTC and 296 controls without cancer) were included in this study. This study was approved by the Institutional Review Board of our tertiary hospital. Informed consent was obtained from all the participants. Given the potential genetic variations between papillary thyroid carcinoma and other thyroid cancer types, this study was limited to patients with papillary thyroid carcinoma to maintain cohort homogeneity.

### 2.2. Selection and Genotyping of Single-Nucleotide Polymorphisms

Genotypes with respect to five *BRCA1* SNPs (rs8176318, rs1799966, rs799917, rs16940, rs1799949) and three *BRCA2* SNPs (rs15869, rs1799943, rs1799955) were determined. These 8 SNPs have been associated with the risk of malignancies of multiple organs in the published literature, and their minor allele frequencies exceeded 10% based on the data provided by National Cancer for Biotechnology Information (http://www.ncbi.nlm.nih.gov/snp, accessed on 7 August 2018) [33,34,35,36,37].

The genotypes were determined using the TaqMan assay method on an ABI prism 7900HT sequence detection system (Applied Biosystems, Foster City, CA, USA), and the used polymerase chain reaction sequences and reaction conditions have been summarized in a previous report [38]. A summary of the SNPs and the probes used in this study is given in Table 1. We also evaluated the potential association of haplotypes with the risk of PTC based on the findings of linkage disequilibrium (LD). Originally, linkage disequilibrium (LD) analysis was conducted for different genes. However, in this study, we analyzed LD based on the assumption that the eight SNPs were located in different positions.

### 2.3. Statistical Analysis

Continuous variables and categorical variables were analyzed using Student’s *t* test and Chi-square test, respectively. We evaluated Lewontin’s D′ (|*D*′|) and the LD coefficient r2 between all the pairs of bi-allelic loci using Haploview v4.2 software, provided by the Broad Institute (Cambridge, MA, USA) (http://www.broadinstitute.org/mpg/haploview, accessed on 7 August 2018) [39]. The haplotypes were inferred using PHASE software (https://stephenslab.uchicago.edu/phase/download.html, accessed on 7 August 2018), and statistical analysis was performed using Statistical Analysis System (SAS) version 9.4 (SAS Inc., Cary, CA, USA) [40]. Logistic regression analyses were performed to compare genotype distributions between the PTC cases and controls, calculating the odds ratio, 95% confidence intervals (CIs), and the corresponding *p*-values. An association analysis was performed using a referent model and 3 alternatives. The referent model compared heterozygotes vs. major homozygotes and minor homozygotes vs. major homozygotes. The co-dominant model compared minor allele homozygotes vs. heterozygotes vs. major allele homozygotes. The dominant model for minor alleles compared minor allele homozygotes plus heterozygotes vs. major allele homozygotes. The recessive model compared minor allele homozygotes vs. heterozygotes plus major allele homozygotes. We considered *p* < 0.05 as statistically significant.

## 3. Results

### 3.1. Genotyping

The demographic characteristics of the participants are summarized in Table 2. There was no significant difference in age between the PTC group and controls. However, the proportion of female patients was higher in the PTC group (75.9% vs. 66.2%, *p* = 0.003). To avoid bias, all the logistic regression analyses were adjusted for age as a continuous covariate and sex as a categorical covariate.

**Figure 1:**
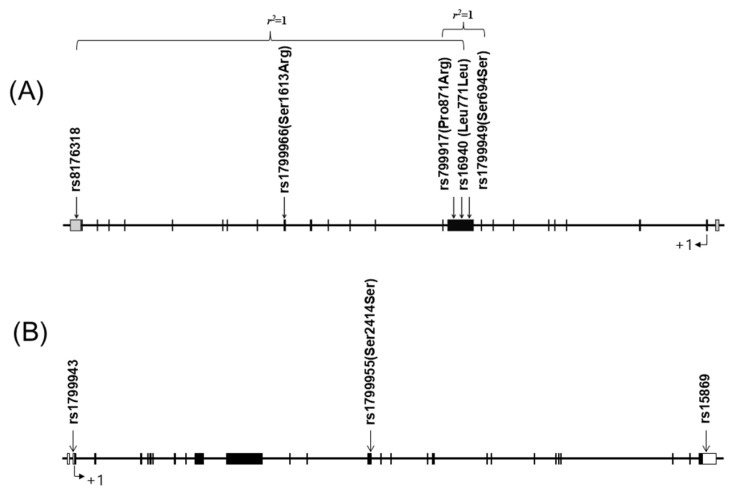
(**A**) Schematic of the BRCA1 DNA repair-associated (*BRCA1*) gene on chromosome 17q21.31. (**B**) Physical map of BRCA2 DNA repair-associated (*BRCA2*) gene on 13q13.1.

The five SNPs of *BRCA1* were all located on chromosome 17q21.31, and the three SNPs of *BRCA2* were located on chromosome 13q13.1 (Figure 1A,B). These eight SNPs were genotyped in 811 study subjects. For the *BRCA1* genes, genotyping for rs8176318, rs1799966, rs799917, rs16940, and rs1799949 was successful in 97.3%, 97.2%, 98.8%, 96.7%, and 99.1% of the subjects, respectively. For the *BRCA2* genes, genotyping for rs1799943, rs1799955, and rs15869 was successful in 98.6%, 98.2%, and 96.1% of the subjects. The quality of genotyping was assessed by duplicate verification in 10% of the samples (rate of concordance in duplicates = 100%).

The alleles, the distribution of genotypes, the frequencies of homozygosity, heterozygosity, and minor alleles, and the tests for deviation from the Hardy–Weinberg equilibrium are listed in Table 3. The distributions of genotypes for all eight genes were not significantly different from the Hardy–Weinberg equilibrium. Minor allele frequency ranged from 0.196 (for rs15869) to 0.406 (rs1799955).

**Figure 2:**
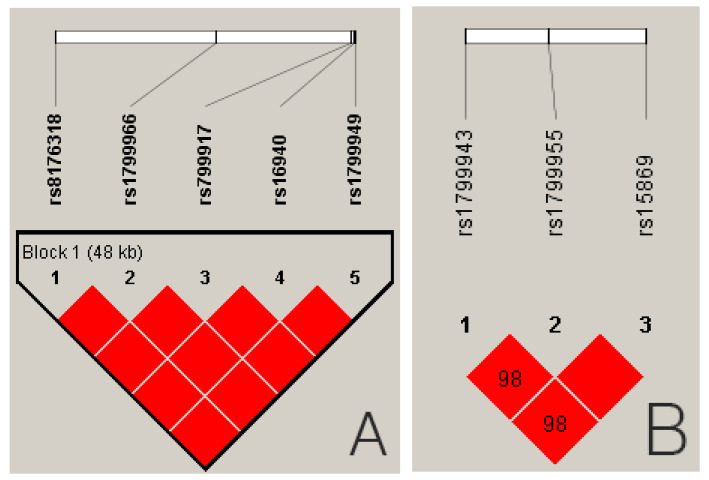
(**A**) Linkage disequilibrium (LD) of single-nucleotide polymorphisms of BRCA1. (**B**) LD of single-nucleotide polymorphisms of BRCA2. The LD was based on the genotypes of the Korean population. In the Haploview program, the D value is expressed as a value between 0 and 100. The value 98 means 0.98. A red diamond without a number represents 1.

An analysis of the linkage disequilibrium revealed that the five *BRCA1* SNPs (rs8176318, rs1799966, rs799917, rs16940, rs1799949) were in complete linkage disequilibrium (all D’= 1.000, Figure 2A). The *r*^2^ values for the *BRCA1* SNPs ranged from 0.985 to 1.000, and the three *BRCA2* SNPs (rs1799943, rs1799955, rs15869) were in strong LD (all D’ ≥ 0.98, Figure 2B). However, the *r*^2^ values were 0.169 between rs1799955 and rs15869, 0.152 between rs1799943 and rs15869, and 0.910 between rs1799943 and rs1799955. We also used the five *BRCA1* SNPs and three *BRCA2* SNPs consecutively to construct haplotypes. This yielded four haplotypes for the *BRCA1* SNPs, of which the two major ones with a frequency exceeding 1% were selected for further statistical analysis. For the SNPs, we constructed six haplotypes, four of which were used for further haplotype analysis. The data for haplotype construction are shown in Appendix A Table A1.

### 3.2. Logistic Regression Analysis of the Association of BRCA1 and BRCA2 Polymorphisms with Thyroid Cancer

**Figure 3:**
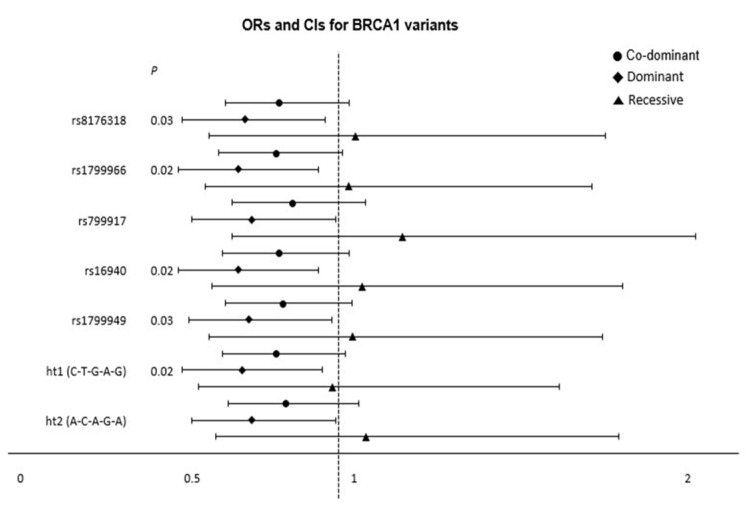
The association results of BRCA1 variants and the risk of PTC.

**Figure 4:**
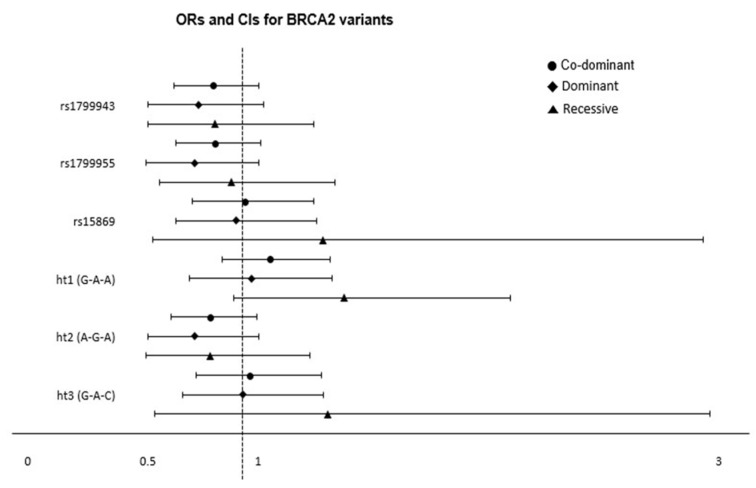
The association results of BRCA2 variants and the risk of PTC.

We performed a logistic regression analysis to evaluate the associations between *BRCA1* and *BRCA2* SNPs and the risk of PTC, adjusting for age and sex (Figure 3 and Figure 4, Table A2, Table A3 and Table A4). Five *BRCA1* SNP genotypes were significantly associated with the risk of PTC. The AC genotype of rs8176318 was associated with a decreased risk of PTC in the referent model (OR = 0.69, 95% CI = 0.51–0.94, *p* = 0.02) and in the dominant model (OR = 0.72, 95% CI = 0.53–0.96, *p* = 0.03). Similarly, the CT genotype of rs1799966 was associated with a decreased risk of PTC in the dominant model (OR = 0.70, 95% CI = 0.52–0.94, *p* = 0.02), and the CC genotype was associated with a decreased risk of PTC in the referent model (OR = 0.67, 95% CI = 0.49–0.91, *p* = 0.01). The AG genotype of rs799917 was significantly associated with the risk of PTC in the referent model (OR = 0.70, 95% CI = 0.52–0.95, *p* = 0.02), and the AG genotype of rs16940 was associated with a decreased risk of PTC both in the referent (OR = 0.67, 95% CI = 0.49–0.91, *p* = 0.01) and dominant (OR = 0.70, 95% = 0.52–0.94, *p* = 0.02) models. Finally, the AG genotype of rs1799949 was associated with a decreased risk of PTC in both the referent (OR = 0.70, 95% CI = 0.52–0.95, *p* = 0.02) and dominant (OR = 0.73, 95% CI = 0.55–0.98, *p* = 0.03) models. Contrarily, there were no significant associations between the genotypes of the three *BRCA2* SNPs and PTC.

Logistic regression with subgroup analyses was performed under the co-dominant, dominant, and recessive models to calculate the ORs (95% CI) and *p*-values. Each plot represents the point estimate of ORs on the x-axis, with 95% CIs represented by error bars. Significant associations are indicated by a *p*-value < 0.05.

### 3.3. Association of BRCA1 and BRCA2 Haplotypes with Thyroid Cancer

The distributions of the haplotype genotypes of *BRCA1* and *BRCA2* and the association of each haplotype with the risk of PTC are summarized in Table A4 and Figure 3 and Figure 4. Haplotype 1 [rs8176318(C)-rs1799966(T)-rs799917(G)-rs16940(A)-rs1799949(G)] ± of *BRCA1* was associated with a significantly decreased risk of PTC in the referent model (OR = 0.69, 95% CI = 0.51–0.93, *p* = 0.02) and the dominant model (OR = 0.71, 95% CI = 0.53–0.95, *p* = 0.02). Haplotype 2 [rs8176318(A)-rs1799966(C)-rs799917(A)-rs16940(G)-rs1799949(A)] ± of *BRCA1* was associated with a decreased risk of PTC in the referent model (OR = 0.71, 95% CI = 0.52–0.96, *p* = 0.03) and the dominant model (OR = 0.74, 95% CI = 0.56–0.99, *p* = 0.04). Haplotypes of *BRCA2* genes were not significantly associated with the risk of thyroid cancer.

## 4. Discussion

The results of this study suggest that *BRCA1* is significantly associated with the pathogenesis of thyroid cancer, while *BRCA2* seems to have little or no association with the risk of PTC. Notably, all five of the evaluated *BRCA1* SNP genotypes were significantly associated with the risk of PTC, and there was no significant association between any of the three *BRCA2* SNPs and PTC. The five *BRCA1* SNPs (rs8176318, rs1799966, rs799917, rs16940, rs1799949) were in complete linkage disequilibrium (all D’= 1.000, *r*^2^ = 0.985~1.000), and the three *BRCA2* SNPs (rs1799943, rs1799955, rs15869) were in strong LD (all D’ ≥ 0.98, *r*^2^ = 0.152~0.910). Two haplotypes of the five *BRCA1* SNP genes and four haplotypes of the three *BRCA2* SNP genes were constructed with frequencies greater than 1%. *BRCA1* haplotype 1 (CTGAG in rs8176318, rs1799966, rs799917, rs16940, and rs1799949) and *BRCA1* haplotype 2 (ACAGA in rs8176318, rs1799966, rs799917, rs16940, and rs1799949) were significantly associated with a decreased risk of PTC. The haplotypes of the *BRCA2* genes were not associated with the risk of PTC. Although the LD analysis was performed on the basis that the SNPs were not in identical locations, caution is needed in interpretation because three SNPs, rs799917, rs16940, and rs1799949, are located in proximity.

*BRCA1* is located at chromosome 17q21 and contains 24 exons that are translated into 1863 amino acids [41]. BRCA1 is involved in signaling DNA damage by forming macromolecular complexes that trigger the repair of double-stranded DNA (dsDNA) breaks with proteins that repair chromosomal damage [42,43]. It is also involved in multiple phases of the cell cycle by interacting with proteins such as p53, p21, BARD1, ATR, and ATM. The deficiency of BRCA1 may result in alterations in the S-phase, G2/M, and cell cycle spindle checkpoints [27]. *BRCA2* rs1799943 is located in an untranslated site of chromosome 13. BRCA2 directly controls the production of RAD51 and repairs DNA breaks through homologous recombination [28]. In *BRCA2*-deficient cells, the sensitivity to radiation is increased, leading to defective dsDNA break repair [44].

Mutations in pathogenic variants of *BRCA1* and *BRCA2* are reported to lead to lifetime risks of developing breast and ovarian cancer, as high as 84 and 45%, respectively [26]. In another study, carriers of impaired *BRCA1* genes had a cumulative risk of breast cancer of 72% by the age of 80, and for *BRCA2*, the corresponding risk was 69% [45]. In addition, the frequency of pathological genetic mutations in *BRCA1* and *BRCA2* in sporadic breast cancers in the South Korean population was found to be 3.1% [46]. It is unclear why breast and ovarian cancers are especially associated with *BRCA* defects since the BRCA proteins are active in all cell types. The association of *BRCA1* and *BRCA2* mutations with thyroid carcinoma has not been well evaluated. Xu et al. examined eight *BRCA1* SNPs in 303 cases of differentiated thyroid carcinoma in the United States [47]; haplotypes that carried the rs799917 variant allele were significantly associated with a decreased risk of thyroid cancer, and the AG/GG genotypes of rs1799950 were associated with a reduced cancer risk, with an adjusted odds ratio of 0.31. These observations agree with our finding that the AG genotype of rs799917 is associated with a reduced risk of PTC. The SNP of rs1799966 was not significantly associated with the risk of differentiated thyroid cancer in that study, unlike in this study. This may be due to the difference in ethnicity of the two populations. A study of a Chinese ethnic population evaluated the association of SNPs in the 3′-UTR double-strand break repair genes *RAD51*, *RAD51B*, *BRCA1* (rs12516, rs8176318), *BRCA2* (rs15869), *XRCC4*, and *XRCC5* with the risk of thyroid cancer in 206 patients with PTC [48]. The CC genotype of *BRCA2* rs15869 was associated with a greater risk of PTC than the AA genotype (OR = 2.595, *p* = 0.031), unlike in our study. Another study evaluating the genetic polymorphisms of the *ATM–CHEK2–BRCA1* axis in 1781 Polish patients with PTC [10] found that *CHEK2* rs17879961 and *BRCA1* rs16941 (OR = 1.6, *p* = 0.005) were risk alleles for PTC. *BRCA1* SNP rs16940 was also associated with the risk of PTC in our study, and we did not evaluate rs16941.

*BRCA1* mutations may also be associated with responses to chemotherapy and cancer survival. One study reported that the polymorphisms of *BRCA1* rs799917 were associated with the response to chemotherapy and the overall survival of Korean lung cancer patients [49]. The TC/CC genotypes of *BRCA1* rs1799966 were associated with a lower risk of death (hazard ratio = 0.617, *p* = 0.028), and the C allele (TC + CC vs. TT) of rs1799966 was associated with a good response to chemotherapy (OR = 0.402, *p* = 0.008) in patients with lung cancer in the Chinese population [50]. This result is interesting because the CT and CC genotypes of rs1799966 were associated with a lower risk of PTC incidence in our study. In another study, the CC genotype of rs1799966 was associated with significantly longer survival in non-small-cell lung cancer patients (Bonferroni-adjusted *p* = 0.012) [36].

The effect of *BRCA1* mutations in thyroid cancer patients is partially understood. The SNPs of *BRCA1* rs1799949 are nonsense mutations and are associated with breast cancer in Taiwan [51]. Another study confirmed a significant association between the rs799917 CT SNP and the risk of gastric cancer (OR = 1.81, *p* = 0.001) and suggested that rs799917C interferes with the interaction between BRCA1 mRNA and miR-638, resulting in reduced BRCA1 expression [52]. A genome-wide association study (GWAS) has evaluated the association between microRNA (miRNA) expression levels and 327 miRNA-related SNPs and between SNPs and colon cancer risk [53]; the AA genotype of *BRCA1* rs8176318 SNP was found to be associated with reduced miRNA expression and also with colon cancer risk (OR 1.31), suggesting that SNP rs8176318 induces thyroid cancer by altering the miRNA–mRNA target interactions. The association of rs8176318 with the risk of PTC may vary between ethnicities. Further investigations are needed to clarify the roles of these genes. *BRCA1* may be associated with thyroid autoimmune disease and affect cancer development through this route. The expression of thyroid hormone receptor beta (TRβ) was significantly higher in breast cancer associated with BRCA1 compared to sporadic breast cancer, the latter not being linked to *BRCA1* (*p* = 0.001) [54]. Furthermore, the expression of TRα alpha in cells with *BRCA1* mutations was linked to a reduction in the 5-year survival rate.

This study has some limitations. First, it was not a randomized study with case-control matching. We adjusted for age and sex so that randomization would reduce any bias. Second, it was not based on whole-genome sequencing and was limited to the polymorphism of the *BRCA* genes, which ideally should not be considered independent of other genes due to the fact that BRCA1 and BRCA2 form stable complexes with partner proteins [43]. As techniques such as whole-genome sequencing improve and their cost drops, genetic analysis using next-generation sequencing should enable the identification of all genetic differences between patients with PTC and controls. Third, the relatively small sample size of both the experimental and control groups is a limitation of this study. Additionally, the number of controls is larger than the case group. This study involved blood collection and genetic testing, and obtaining consent from a large number of control group participants was limited due to cultural considerations in the Korean population. A large-scale study is necessary through multicenter research in the future. Fourth, additional SNPs besides *BRCA1* and *BRCA2* should also be analyzed to evaluate the non-random association of each SNP due to the proximity of BRCA1 SNPs analyzed in this study [55].

Since there were few cases with recurrence, we could not analyze the genetic features related to recurrence. Future research is needed to identify genes associated with thyroid cancer recurrence. Compared to well-established mutations, the interpretation of the direct causal relationship between polymorphisms and cancer requires caution. Further investigations should be performed to distinguish between association and direct causation. Nevertheless, many recent studies have demonstrated that SNPs are associated with cancer prognosis and can predict treatment response [56,57,58,59]. Although pathogenic variants of *BRCA1* may have potentially caused thyroid cancer, it is possible to interpret that thyroid cancer was found in individuals with pathogenic *BRCA1* mutation. Since SNPs include regions that are not translated into proteins, additional protein analysis is necessary for meaningful interpretation. Potential limitations of study evaluating the association of SNPs and cancer risk include false positive associations, difficulty of pinpointing the casual variant within a haplotype, and the possibility of overlooking rare variants, which may have more significant effects.

Further studies including miRNA, proteomic, and genomic biomarker analyses will enhance the comprehension of PTC etiopathogenesis [60].

## 5. Conclusions

Our findings suggest that the five *BRCA1* SNPs (rs16940, rs799917, rs1799949, rs1799966, rs8176318) and haplotypes of *BRCA1* contribute to the susceptibility of developing thyroid cancer. There was no association between *BRCA2* and the risk of PTC. However, it is necessary to be cautious in interpreting the association between SNPs and the risk of thyroid cancer as evidence that SNP variations directly cause cancer. Further functional studies with a larger number of individuals, including diverse ethnicities and other genes related to the genetic pathway beyond BRCA, are needed to establish the role of *BRCA1* in PTC.

## Figures and Tables

**Table 1:** Probes for *BRCA1* and *BRCA2* polymorphisms.

Gene	SNPID	Assay-on-Demand ID	Company
*BRCA1*	rs8176318	C_3178688_10	Thermo Fisher Scientific, Waltham, MA, USA
	rs1799966	C_2615208_20	
	rs799917	C_2287943_10	
	rs16940	C_11415267_10	
	rs1799949	C_2615193_10	
*BRCA2*	rs1799943	C_3070446_10	
	rs1799955	C_7605612_10	
	rs15869	C_807118_10	

**Table 2:** Demographics of patients with papillary thyroid carcinoma and controls.

Variable	PTC ^a^ Cases (*n* = 515)	Controls (*n* = 296)	*p* Value
Age (mean ± SD)	47.43 ± 12.11	47.22 ± 14.20	0.829
Age, Male (mean ± SD)	46.88 ± 12.91	48.10 ± 13.31	0.489
Age, Female (mean ± SD)	47.61 ± 11.86	46.78 ± 14.65	0.458
Gender			0.003
Male	124 (24.1%)	100 (33.8%)	
Female	391 (75.9%)	196 (66.2%)	

^a^ Papillary thyroid carcinoma.

**Table 3:** Allele distributions and Hardy-Weinberg equilibria of *BRCA1* and *BRCA*2 polymorphisms.

Gene	Loci	Allele	Major Homozygosity	Heterozygosity	Minor Homozygosity	Total	MAF ^a^	HWE ^b^
*BRCA1*	rs8176318	C>A	402	322	65	789	0.286	0.963
	rs1799966	T>C	398	324	66	788	0.289	0.996
	rs799917	G>A	410	327	64	801	0.284	0.915
	rs16940	A>G	393	326	65	784	0.291	0.821
	rs1799949	G>A	409	330	65	804	0.286	0.891
*BRCA2*	rs1799943	G>A	297	379	124	800	0.392	0.865
	rs1799955	A>G	282	381	133	796	0.406	0.822
	rs15869	A>C	503	246	30	779	0.196	0.991

^a^ Minor allele frequency; ^b^ Hardy–Weinberg Equation; ^b^ *p*-value for the test for Hardy–Weinberg equilibrium.

## Data Availability

The original contributions presented in this study are included in the article. Further inquiries can be directed to the corresponding authors.

## Appendix A

**Table A1:** Haplotypes of *BRCA1* and *BRCA2* polymorphisms.

Genes	Haplotype	rs8176318	rs1799966	rs799917	rs16940	rs1799949	Frequency
BRCA1	Ht ^a^1	C	T	G	A	G	0.711
	Ht2	A	C	A	G	A	0.285
	others	-	-	-	-	-	0.003
		rs1799943	rs1799955	rs15869			
BRCA2	Ht1	G	A	A			0.393
	Ht2	A	G	A			0.390
	Ht3	G	A	C			0.196
	Ht4	G	G	A			0.018
	others	-	-	-			0.003

^a^ Haplotype.

**Table A2:** Associations of *BRCA1* SNPs with the risk of papillary thyroid carcinoma.

Loci	Genotype	Genotype Distribution		Referent		Co-Dominant		Dominant		Recessive	
		PTC ^a^	Control	OR ^b^ (95% CI ^c^)	*p*	OR (95% CI)	*p*	OR (95% CI)	*p*	OR (95% CI)	*p*
rs8176318	CC	271 (53.8%)	131 (46.0%)	1							
	AC	190 (37.7%)	132 (46.3%)	0.69 (0.51–0.94)	0.0 2 *	0.82 (0.66–1.03)	0.10	0.72 (0.53–0.96)	0.03 *	1.05 (0.61–1.80)	0.86
	AA	43 (8.5%)	22 (7.7%)	0.91 (0.52–1.59)	0.74						
rs17-99966	TT	272 (53.5%)	126 (45.0%)	1							
	CT	192 (37.8%)	132 (47.1%)	0.87 (0.50–1.53)	0.64	0.81 (0.64–1.01)	0.07	0.70 (0.52–0.94)	0.02 *	1.03 (0.60–1.76)	0.93
	CC	44 (8.7%)	22 (7.9%)	0.67 (0.49–0.91)	0.01 *						
rs799917	GG	275 (53.7%)	135 (46.7%)	1							
	AG	193 (37.7%)	134 (46.4%)	0.70 (0.52–0.95)	0.02 *	0.86 (0.68–1.08)	0.19	0.74 (0.56–0.99)	0.05	1.19(0.68–2.07)	0.54
	AA	44 (8.6%)	20 (6.9%)	1.05 (0.59–1.86)	0.88						
rs16940	AA	269 (53.1%)	124 (44.8%)	1							
	AG	194 (38.3%)	132 (47.7%)	0.67 (0.49–0.91)	0.01 *	0.82 (0.65–1.03)	0.08	0.70 (0.52–0.94)	0.02 *	1.07 (0.62–1.85)	0.81
	GG	44 (8.7%)	21 (7.6%)	0.91 (0.51–1.60)	0.74						
rs1799949	GG	274 (53.5%)	135 (46.2%)	1							
	AG	195 (38.1%)	135 (46.2%)	0.70 (0.52–0.95)	0.02 *	0.83 (0.66–1.04)	0.11	0.73 (0.55–0.98)	0.03 *	1.04 (0.61–1.79)	0.88
	AA	43 (8.4%)	22 (7.5%)	0.91 (0.52–1.60)	0.75						

^a^ Papillary thyroid carcinoma. ^b^ Odds ratio. ^c^ Confidence interval. All *p*-values are for logistic regression analysis, adjusted for age and gender. * *p* < 0.05.

**Table A3:** Associations of *BRCA2* SNPs with the risk of papillary thyroid carcinoma.

Loci	Genotype	Genotype Distribution		Referent		Co-Dominant		Dominant		Recessive	
		PTC ^a^	Control	OR ^b^ (95% CI ^c^)	*p*	OR (95% CI)	*p*	OR (95% CI)	*p*	OR (95% CI)	*p*
rs1799943	GG	200 (39.0%)	97 (33.8%)	1							
	AG	237 (46.2%)	142 (49.5%)	0.81 (0.59–1.12)	0.21	0.87 (0.70–1.07)	0.19	0.81 (0.59–1.09)	0.16	0.88 (0.59–1.31)	0.52
	AA	76 (14.8%)	48 (16.7%)	0.79 (0.51–1.23)	0.30						
rs1799955	AA	191 (37.5%)	91 (31.8%)	1							
	AG	236 (46.3%)	145 (50.7%)	0.77 (0.56–1.07)	0.12	0.88 (0.71–1.08)	0.23	0.79 (0.58–1.07)	0.12	0.95 (0.64–1.40)	0.78
	GG	83 (16.3%)	50 (17.5%)	0.82 (0.53–1.27)	0.37						
rs15869	AA	316 (64.6%)	187 (64.5%)	1							
	AC	152 (31.1%)	94 (32.4%)	0.94 (0.68–1.28)	0.68	1.01 (0.78–1.31)	0.93	0.97 (0.71–1.32)	0.84	1.35 (0.61–3.00)	0.45
	CC	21 (4.3%)	9 (3.1%)	1.33 (0.59–2.98)	0.48						

^a^ Papillary thyroid carcinoma. ^b^ Odds ratio. ^c^ Confidence interval. All *p*-values are for logistic regression analysis, adjusted for age and gender.

**Table A4:** Associations of BRCA1 and BRCA2 haplotypes with the risk of papillary thyroid carcinoma.

Gene	Loci	Genotype	Genotype Distribution		Referent		Co-Dominant		Dominant		Recessive	
			PTC ^a^	Control	OR ^b^ (95% CI ^c^)	*p*	OR (95% CI)	*p*	OR (95% CI)	*p*	OR (95% CI)	*p*
BRCA1	ht ^d^1 (C-T-G-A-G)	-/-	276 (53.6%)	135 (45.6%)	1							
		ht1/-	195 (37.9%)	137 (46.3%)	0.69 (0.51–0.93)	0.02 *	0.81 (0.65–1.02)	0.07	0.71 (0.53–0.95)	0.02 *	0.98 (0.58–1.66)	0.95
		ht1/ht1	44 (8.5%)	24 (8.1%)	0.85 (0.49–1.47)	0.56						
	ht ^d^2 (A-C-A-G-A)	-/-	276 (53.6%)	138 (46.6%)	1							
		ht2/-	195 (37.9%)	136 (45.9%)	0.71 (0.52–0.96)	0.03 *	0.84 (0.67–1.06)	0.14	0.74 (0.56–0.99)	0.04 *	1.08 (0.63–1.84)	0.79
		ht2/ht2	44 (8.5%)	22 (7.4%)	0.95 (0.54–1.65)	0.84						
BRCA2	ht ^e^1 (G-A-A)	-/-	193 (37.5%)	113 (38.2%)	1							
		ht1/-	229 (44.5%)	144 (48.6%)	0.94 (0.69–1.29)	0.71	1.12 (0.91–1.38)	0.28	1.04 (0.77–1.39)	0.81	1.44 (0.96–2.16)	0.07*
		ht1/ht1	93 (18.1%)	39 (13.2%)	1.38 (0.88–2.14)	0.15						
	ht ^e^2 (A-G-A)	-/-	201 (39.0%)	99 (33.4%)	1							
		ht2/-	241 (46.8%)	149 (50.3%)	0.80 (0.58–1.10)	0.17	0.86 (0.69–1.06)	0.15	0.79 (0.59–1.07)	0.13	0.86 (0.58–1.29)	0.48
		ht2/ht2	73 (14.2%)	48 (16.2%)	0.77 (0.50–1.20)	0.25						
	ht ^e^3 (G-A-C)	-/-	332 (64.5%)	192 (64.9%)	1							
		ht3/-	161 (31.3%)	95 (32.1%)	0.96 (0.70–1.31)	0.81	1.03 (0.80–1.34)	0.81	1.00 (0.74–1.35)	0.98	1.37 (0.62–3.03)	0.43
		ht3/ht3	22 (4.3%)	9 (3.0%)	1.36 (0.61–3.02)	0.44						

^a^ Papillary thyroid carcinoma, ^b^ odds ratio, ^c^ confidence interval, ^d^ haplotype. Each haplotype consisted of rs8176318, rs1799966, rs799917, rs16940, and rs1799949. ^e^ Each haplotype consisted of rs1799943, rs1799955, and rs15869. All *p*-values are for logistic regression analysis adjusted for age and gender. * *p* < 0.05.
